# A new matrix for scoring the functionality of national laboratory networks in Africa: introducing the LABNET scorecard

**DOI:** 10.4102/ajlm.v5i3.498

**Published:** 2016-10-31

**Authors:** Pascale Ondoa, Tjeerd Datema, Mah-Sere Keita-Sow, Jean-Bosco Ndihokubwayo, Jocelyn Isadore, Linda Oskam, John Nkengasong, Kim Lewis

**Affiliations:** 1 Amsterdam Institute for Global Health and Development (AIGHD), Department of Global Health, Academic Medical Center, Amsterdam, the Netherlands; 2 Royal Tropical Institute Biomedical Research (KIT), Amsterdam, the Netherlands; 3 DATOS, Amsterdam, the Netherlands; 4 African Society of Laboratory Medicine (ASLM), Addis Ababa, Ethiopia; 5 World Health Organization Regional Office for Africa, Brazzaville, Republic of Congo; 6 Association of Public Health Laboratories, Silver Spring, Maryland, United States; 7 Division of Global HIV and TB, International Laboratory Branch, US Centers for Disease Control and Prevention, Atlanta, Georgia, United States

## Abstract

**Background:**

Functional national laboratory networks and systems are indispensable to the achievement of global health security targets according to the International Health Regulations. The lack of indicators to measure the functionality of national laboratory network has limited the efficiency of past and current interventions to enhance laboratory capacity in resource-limited-settings.

**Scorecard for laboratory networks:**

We have developed a matrix for the assessment of national laboratory network functionality and progress thereof, with support from the African Society of Laboratory Medicine and the Association of Public Health Laboratories. The laboratory network (LABNET) scorecard was designed to: (1) Measure the status of nine overarching core capabilities of laboratory network required to achieve global health security targets, as recommended by the main normative standards; (2) Complement the World Health Organization joint external evaluation tool for the assessment of health system preparedness to International Health Regulations (2005) by providing detailed information on laboratory systems; and (3) Serve as a clear roadmap to guide the stepwise implementation of laboratory capability to prevent, detect and act upon infectious threats.

**Conclusions:**

The application of the LABNET scorecard under the coordination of the African Society of Laboratory Medicine and the Association of Public Health Laboratories could contribute to the design, monitoring and evaluation of upcoming Global Health Security Agenda-supported laboratory capacity building programmes in sub Saharan-Africa and other resource-limited settings, and inform the development of national laboratory policies and strategic plans. Endorsement by the World Health Organization Regional Office for Africa is foreseen.

## Background on laboratory systems in Africa

In spite of current funding and partnership opportunities to strengthen laboratory capacity in resource-limited settings, poor access to quality laboratory testing continues to lead to misdiagnosis, inappropriate treatment,^1^ increased morbidity and mortality, and an inability to determine the true prevalence of diseases.^2^ Unreliable test results generate mistrust in laboratory services by clinical staff, aggravating the misuse of drugs such as antibiotics and causing undue cost to the patients.^3^ In addition, delays in laboratory testing confirmations complicate the public health response and impede the control of epidemic diseases.^4,5,6^

A national laboratory network can be defined as a collaborative group including all (public, private and private not-for-profit) medical laboratories within a country. The networks are typically organised in three to five-tiered structures of testing facilities operating under common principles and procedures with tier-specific roles, responsibilities and functions.^7^ The national laboratory network supports the entire health system in accessible, high-quality and efficient testing for individual patient care and public health needs.^7^ Peter et al. highlight that the proper functioning of the national laboratory networks requires central management and direction through policies, regulatory oversight and coordination of operational functions,^8^ but these components are missing in many networks. Because the laboratory is a key component of the health system and is recognised as one of the six essential core functions of public health identified by the US Centres for Disease Control and Prevention, for which strengthening would have the widest influence on the health system,^9^ it is essential to aid countries to build laboratory capacity to detect, report and respond to public health events. In the early 2000s, the combined efforts of global health partners (US Centres for Disease Control and Prevention, Clinton Health Access Initiative, Global Alliance for Vaccines and Immunization, Global Fund), national and international institutions (World Health Organization [WHO] and governments) have led to several ambitious programmes to strengthen laboratory systems in sub-Saharan Africa. This momentum is illustrated by the numerous landmark meetings on laboratory policy that took place between 2008 and 2012,^10,11^ and which provided a framework for laboratory system strengthening and development in sub-Saharan Africa. Other key accomplishments are the creation of the World Health Organization Regional Office for Africa based Stepwise Laboratory Improvement Towards Accreditation (SLIPTA),^10^ the development of the Strengthening Laboratory Management Toward Accreditation (SLMTA)^12^ training programme, and the launch of the African Society of Laboratory Medicine (ASLM), three important initiatives for the improvement of laboratory systems in Africa.^11^

In the same period, substantial funding has been dedicated to integrating laboratory services, developing laboratory policy and strategic planning for tiered laboratory networks, implementing quality system improvement schemes, and increasing the laboratory workforce. Despite the efforts to strengthen laboratory systems and networks and the remarkable level of resources made available, serious gaps remain. Such insufficiencies include shortages in skilled and trained professionals, inadequate and poorly-maintained infrastructures and equipment, inconsistent supply of reagents and consumables, lack of clear national policies and poor leadership. In practice, laboratory diagnostics are unavailable for most fevers^13^ and accurate laboratory results are lacking to support evidence-based treatment of diseases, surveillance and outbreak investigations. These deficiencies were dramatically exposed during the recent Ebola epidemic, which claimed 11 323 victims in Sierra Leone, Guinea and Liberia,^14^ and underscore the lack of preparedness of many countries to detect, respond to and prevent health threats. In the context of increasing international travel and trade and of emerging and re-emerging infectious diseases, dysfunctional laboratory systems and networks of Africa constitute a serious barriers to reducing mortality and morbidity and to achieving International Regulation (IHR) 2005 goals for international and collective response to outbreaks. Failure to address the chronic weaknesses of African laboratory systems represents an impediment to achieve several of the United Nations 2030 sustainable development goals.^15^

## Determinants of remaining gaps in national laboratory network capabilities

The causes of persisting deficiencies of the laboratory systems and network are multifactorial and include: the influence of vertical programmes;^16^ insufficient strengthening of general national laboratory networks;^8^ poorly-implemented laboratory policies and improvement strategies;^17^ and a lack of clear indicators to measure the status of laboratory systems and networks^18,19^ in current standardised tools available to assess laboratories. These tools are either facility-oriented (e.g., WHO Laboratory Assessment Tool facility assessment,^20^ WHO SLIPTA^21^) and/or address individual aspects of laboratory system,^22^ and/or are narrowed to one disease,^23^ and/or are not specific to laboratories,^24^ and/or measure absence of key components without sufficiently characterising the discrete levels of laboratory network capabilities to guide performance improvement initiatives.^25^ Furthermore, system or network standards may not be adequately defined to set actionable objectives.^25^ The inability to assess benchmarks of laboratory systems and networks performance hides the reality of the problem, weakens advocacy efforts and makes progress impossible to measure.

Collectively, these observations call for the development of a novel standardised framework capable of capturing metrics relevant to the laboratory network performance and measuring progress toward a more comprehensive set of laboratory systems standards.

## Current normative standards for national medical laboratory networks

Normative standards, directives and recommendations guiding the development of national laboratory systems and networks include: requirements for public, international and global health;^26^ access to primary healthcare; disease control at the human, animal and ecosystem interface;^27,28^ surveillance of diseases;^29^ and directives for sustainable, integrated and quality laboratory services.^30^ In addition, the recently-launched Global Health Security Agenda (GHSA) provides additional resources and recommendations to accelerate the implementation of the IHR 2005, especially in terms of infectious disease control. The GHSA objectives specifically underscore national laboratory systems and laboratory-based disease surveillance systems, comprehensively cover essential public health and clinical functions and highlight the need for medical laboratory services to collaborate closely with other sectors within the Ministry of Health and other agencies.^31^ Essential laboratory requirements per key normative standards and regulation are provided in Table 1.

**TABLE 1:** Components and targets (stage 5) per core capabilities.

Nine overarching core capability-associated components	Overall targets (representing overall stage 5)
**1-Legal and regulatory framework** National policy and plans Legislation Governance Finances	**Target 1:** The country has a fully endorsed legal and regulatory framework that enables management of a national laboratory system and compliance with the International Health Regulations (IHR) and that organises and controls all public and private laboratory services under the OneHealth concept, with sufficient dedicated funding available.
**2-Structure and organisation of the laboratory networks** Tiered laboratory network Coordination & management	**Target 2:** Sustainable, rational and efficient tiered laboratory network(s), provides integrated, essential, quality diagnostic services for patient care and public health. The network for human health is coordinated by a national reference or public health laboratory and includes community laboratory services. It regroups all laboratories from public and private sector in the country and collaborates with other laboratories under the OneHealth concept involved in Public Health. Each tier of the laboratory network has clearly defined terms of reference, and is adequately supervised.
**3-Network coverage and rapid response** Tiered network coverage Rapid response and preparedness Sample referral system	**Target 3:** The national laboratory network(s) is fully integrated and accessible to the whole population throughout the country. Diagnostic services are accessible to control the safety of persons and good at all international entry points. The laboratory surge capacity supports the rapid response to health emergencies. Comprehensive system is in place for the referral of biological specimens (including sentinel sites or unusual samples from the routine) upstream to the appropriate level (also internationally).
**4-Laboratory information (management systems)** Data collection Data analysis and sharing Surveillance/Epidemiology Reporting Security and confidentiality of information	**Target 4:** Inter-operable and interconnected electronic recording and reporting systems are in places that generate reliable data that are monitored and analysed in real time for potential health threats that may require public health action. These systems comply with international standards to allow the rapid exchange of information at national and international level. A laboratory information management system also provides up to date information about the status of the laboratories and is linked to the Health Management Information System of the country.
**5-Infrastructure** Laboratory facilities Supply chain management Equipment	**Target 5:** Laboratory testing in performed in dedicated and modern laboratories fulfilling (inter)national standards of (bio) safety (see Target 7). Testing is performed with state-of-the-art and well maintained equipment using a regular supply of quality reagents and consumables and standardised testing methods throughout the country.
**6-Human Resources** Education and training Staffing Human resources development strategy	**Target 6:** Adequate numbers of competent, well-trained and motivated technical and managerial staff are available at all levels of the laboratory system.
**7-Quality of the laboratory system** Quality Assurance Quality Management System Accreditation	**Target 7:** All tiers of the laboratory network provide high quality services with accurate and reliable results. Tiered external quality assurance programmes are organized by the networks to assess the quality of the services at all levels, including the quality of point of care testing outside the laboratory. A system of national certification is in place for all public and private laboratories. Laboratories at the highest tier of the laboratory network are accredited according to (inter)national standards (ISO15189 or ISO17025, depending on the laboratory).
**8-Biosafety and biosecurity** Biosafety manual National biosafety and biosecurity system Specimen storage Waste management	**Target 8:** Laboratory biosafety and biosecurity is an explicit part of the whole-of-government national biosafety and biosecurity system. Testing is performed in a manner and in facilities that guarantee safety for the staff, the customers, the community and the environment. Sufficient materials, means and skills are available throughout the system to ensure safe and secure procurement, handling, storage, transportation and disposal of samples and materials, both in routine as well as in emergency circumstances. Biobanking is nationally owned and organised according to a standard of biosafety and biosecurity.
**9-Priority diseases** Prioritisation Testing Antimicrobial resistance	**Target 9**: The country has prioritised diseases and pathogens and developed plans and protocols for the prevention, detection and response, which are adequate for all categories of pathogens. The laboratory network has the capacity to test all priority pathogens, including antimicrobial resistance and zoonotic diseases, in collaboration with laboratories from the animal health and environmental sectors. There is a comprehensive system is in place for the referral of biological specimens (including sentinel sites or unusual samples from the routine) upstream to the appropriate level.

Targets describing the international standard and corresponding to stage 5 were formulated for each core capability. Stepwise improvement from stage 0 (absence of key attribute) to stage 5 was subsequently described for each core capability, broken down into components and indicators. Hence, the targets represent the summary of score 5 for all indicators describing a core capability.

Conceivably, the degree of availability of resources (material, human and technical) and capacity to conduct the necessary improvements toward all key normative standards correlates with the degree of the national laboratory network’s capability to carry out essential public health and clinical functions.

## Aim

GHSA-supported programmes strive to support the implementation of health security preparedness as a way to comprehensively strengthen laboratory capacity for all essential public health and clinical functions.^31^ An important step to further advance public health laboratory systems toward health security is to conduct objective evaluations of each country’s national laboratory network preparedness.^32^ Such assessments serve as a basis to develop targeted enhancement plans and to measure progress of the national laboratory network capability to support the prevention, detection and response to health threats. To such end, the African Society for Laboratory Medicine (ASLM) commissioned the development of a standardised methodology to assess progress and changes in national laboratory networks’ performance toward Global Health Security targets.

The assessment tool was designed through a collaborative effort between the Amsterdam Institute for Global Health and Development, the Association of Public Health Laboratories (APHL) and the Royal Tropical Institute – Biomedical Research of the Netherlands (currently DATOS).

### Terms of reference: the LABNET scorecard

The desired features for a standardised tool to assess national laboratory network functionality against current normative standards include:

- The overarching assessment of a full set of core capabilities required to achieve all key laboratory standards of global health security.
- Clear indicators describing the maturation of each core capability according to a linear scale of increasing functionality.
- A practical roadmap of what needs to be implemented for the country to achieve next levels of functionality for each core capability. This could serve as the basis to define targeted improvement strategies at regional or at country level that can leverage advancement toward global health security targets and/or the development of well-informed national laboratory policies and plans.
- The possibility to provide a quick visual representation of national laboratory networks functionality within a country and across countries, which can serve advocacy efforts toward high level policymakers.

The capability maturation model (CMM)^33^ was selected as the most appropriate format to design the national laboratory network assessment tool. CMM is a structured and sequential approach to evaluation^34^ which provides a matrix describing an evolutionary path from ad hoc, chaotic processes to mature, disciplined processes. The generic framework can be applied to the assessment of any system^35,36^ with a scorecard describing five levels of capability, generally used to evaluate system maturity.^34^

## Design of the scorecard

The scorecard for national laboratory network functionality (hereafter referred to as the LABNET scorecard) was designed as a CMM and began with: (1) the definition of the laboratory network core capabilities necessary to conduct clinical and public health functions supporting global health security targets; and (2) the description of the maturation stages for each capability in such a way that improvements at each stage provide the foundation on which to build improvements undertaken at the next stage. Hence, the scorecard can identify deficiencies and guide advancement of the laboratory network. Core capabilities are typically described by one to four criteria or components against which indicators are developed.

## Selection of core capabilities and components

Core capabilities were defined as the overarching functions of the country national laboratory network to detect, assess, notify and respond to health events in accordance with the main normative standards guiding the development of national laboratory network – IHR; GHSA; the WHO guidance to establish national health laboratory systems;^37^ the WHO Integrated Disease Surveillance and Response;^38^ the WHO global strategy for the containment of antimicrobial resistance (AMR);^39^ and the WHO regional guide for establishing laboratory-based surveillance of AMR^40^ – under the One Health concept^27^ and following the recommendations of the Maputo Declaration for strengthening laboratory systems in resource-poor settings.^30^ Fifty essential functions described in at least two normative standards or guiding documents were listed and grouped into nine main themes, which served as core capabilities to be assessed for the national laboratory network CMM.

One to three components best describing each core capability were drawn from the list of most recurrent functions described in normative documents. The nine core capabilities identified across the main relevant guidelines, their corresponding components and targets are shown in Table 1.

## Development of indicators and definition of capability levels

Indicators characterising the functionality of each component were developed in the form of questions, to measure the maturity of the system along three key dimensions:

- **Inputs:** including infrastructure, commodities, technology, equipment, people, legislation, policies, and finances.
- **Processes:** including networking, procedures for processes, implementation, expansion, coverage, quality and integration of laboratory network services.
- **Measurement:** including targets, indicators, output, outcomes, cost-efficiency, data reporting systems, data collection and data use to improve the quality of the laboratory network.

**FIGURE 1:**
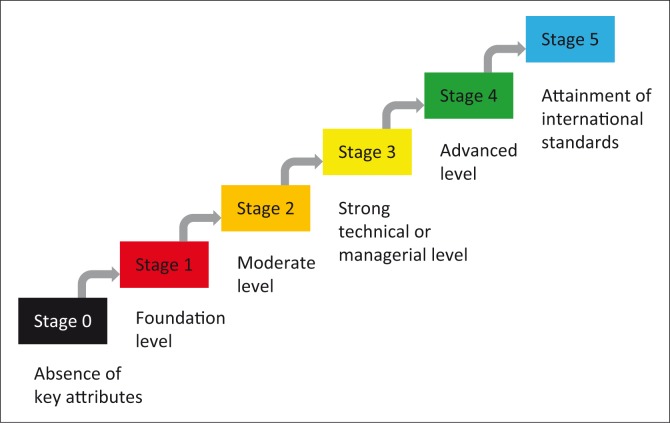
Maturation stages proposed for each core capability (or function) of the national laboratory network.

**FIGURE 2:**
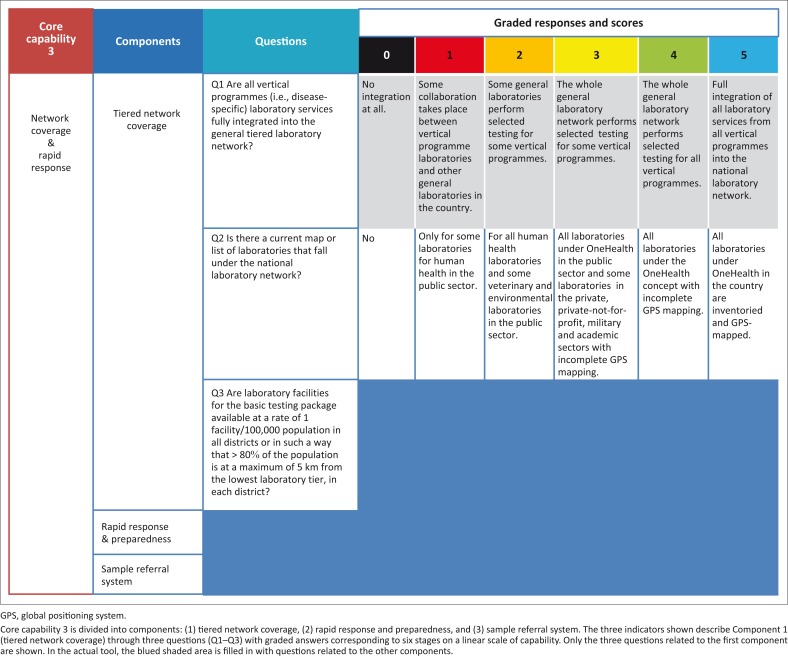
Example organisation of core capabilities, components and indicators on LABNET scorecard.

The definition of capability levels in relation to input, process and measurement is provided in Figure 1. Given the anticipated weaknesses of laboratory networks in some areas, a stage 0 was incorporated to match situations in which no key attributes are present in a given core capability. In order to obtain sufficient granularity, each component was broken down in to one to four questions with responses grading from 0 to 5, describing the situation along the capability maturation scale. The measurements are mostly descriptive and qualitative, but some incorporate a quantitative dimension. The example of core capability 3 (coverage and rapid response) and its associated components and indicators broken down into questions with graded responses is shown in Figure 2.

## Purpose of the LABNET scorecard assessment

The LABNET scorecard assesses a country’s national laboratory network functionality to support the implementation of the IHR 2005 and to reach the GHSA targets of preventing, detecting and responding to infectious disease threats. A regional and international evaluation process is foreseen in GHSA target countries in Africa and Asia, under the coordination of ASLM and APHL, two organisations mandated to implement the GHSA, as a way to accelerate the achievement of the IHR 2005. The LABNET scorecard assessment is voluntary and is applied through a series of external evaluations that will allow a country to measure the progress of the national laboratory network towards the health security targets. The first evaluation will provide a baseline measurement of the national laboratory network capabilities and guide the design and implementation of laboratory strengthening initiatives. Subsequent evaluation(s) will measure progress made and ensure improvements in capacity are sustained.

## Scoring system and options for data analysis

Based on the current country situation, each question receives a unique score from 0 (key attribute(s) completely absent) to 5 (key attribute(s) compliant with international standards), describing the level of maturity. Key definitions and rationale behind the questions are incorporated into the scorecard to facilitate the scoring process. Without achievement of all attributes at prior capability level, the national laboratory network cannot achieve the next level. The responses should be supported by documentation whenever possible. The score attributed to each indicator can be used in two ways, as described below.

### Percentage-based scoring system

**FIGURE 3:**
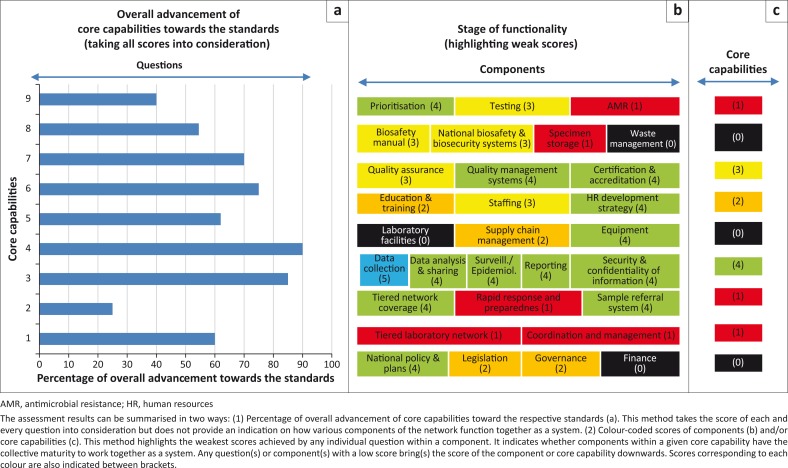
Summary of country results using percentages and color-codes.

A ratio can be calculated dividing the total number of points scored for all questions within a core capability by the maximum possible score (total number of questions x 5). The percentages are useful in estimating the overall degree of advancement of each core capability toward the standards (Figure 3A). However, the percentages provide little information on the extent to which the various components within a core capability mature and work together towards a functional laboratory network.

### Color-scoring system of component and core capabilities

According to the CMM methodology, up to two additional layers of scores can be calculated for each of the component and the core capabilities.

Each component can be assigned a score, which corresponds to the lowest score achieved by any question within that component. This tends to bring the overall results down the scale of maturity, while allowing the identification of critical weaknesses that prevent the system from achieving higher stages. Similarly, each core capability can be assigned a score, which corresponds to the lowest score achieved by any component within that core capability (Figure 3B). Any core capability or component with a low score offers the opportunity for drilling down to identify specific component(s) and/or specific question(s) which have not been adequately addressed by the country and require follow-up actions. Color-coded graphs allow for a straightforward visual representation of scoring (figures 3B and 3C).

## Assessment process

In order to ensure the optimal standardisation of the assessment process, a pool of Francophone and Anglophone assessors from Africa and the United States were trained to use the LABNET scorecards in a joint ASLM and APHL effort and a detailed scorecard instruction manual was developed (supplementary material). The first stage of the evaluation is the country self-reporting for all the indicators across the nine core capabilities of the LABNET scorecard. A national committee led by the country contact point for the national laboratory network and including a wide representation of stakeholders under the One Health concept will complete the self-evaluation. A list of typical committee members is provided in Box 1.

**BOX 1:** List of national committee members.

• MOH laboratory network representatives including: ◦ Laboratory Services Director ◦ Global Health Security Agenda focal person ◦ Laboratory Quality Unit ◦ Biosafety and Biorisk Unit ◦ Procurement and Supply Management Unit ◦ Finance Unit ◦ Human Resource Unit ◦ Environment and Infection Control Unit ◦ Planning Unit ◦ Statistics Unit
• Private laboratory sector
• Education sector
• Veterinary sector
• Agricultural sector
• Representation for the laboratory tiers as appropriate
• Technical international partners

A LABNET scorecard evaluation team comprising three international experts, fluent in the country language, reviews the information prior to a one-week visit to the country. The visit is comprised of an initial two-day workshop during which the evaluation team members review and validate the pre-filled data with the national committee, gather information explaining the scores and collect documented evidence. During the next three days, the evaluation team visits representative laboratory facilities at each tier level. The visits also include key departments, such as the national health data unit and the central procurement and distribution facility for laboratory consumables. Site visits aim to verify information provided at the central level and are not structured laboratory facility assessments.

After completing the country visit, the evaluation team drafts a report to identify scores levels for each indicator, and to describe the status of each component and core capability. Based on the explanations provided to justify the scores assigned to each indicator, the report also includes a root-cause analysis, supporting the evidence-based selection of strategies with highest leverage potential for comprehensive laboratory improvement. Upon validation by ASLM and/or APHL, the report is shared with the host country and with other relevant stakeholders, as permitted by the country.

## Validation of the LABNET scorecard: lessons learnt

Following the initial development phase, the LABNET scorecard underwent revision, consolidation and validation, scrutinising the relevance, order of the questions, logic of scores, and clarity of phrasing. In total, more than 40 persons provided input on the LABNET scorecard during:

- the Freetown meeting organised by ASLM during a breakout session attended by 25 experts (Freetown, Sierra Leone, October 2015);
- a meeting of APHL US senior laboratory experts on system assessment of national laboratory network (Silver Spring, MD, US, February 2016); and
- a meeting of 16 African senior laboratory facility and laboratory system assessors (Dakar, Senegal, February 2016).

The feasibility of using the revised and consolidated version of the LABNET scorecard was tested through pilot assessments in Uganda (December 2015) and Tanzania (February 2016). The key lessons learnt were multifold:

1. Getting country buy-in is crucial. A series of interactions between the host country and the implementing coordinating partners needs to take place before the assessment so that the importance of the evaluation and its complementarities with other ongoing assessments is clearly understood. This preparatory work also guides the country in collecting all necessary evidence supporting the scoring beforehand.
2. The LABNET scorecard assessment is a pedagogic tool to introduce system-thinking to stakeholders not previously familiar with the process. Highlighting root-cause of interrelated problems helped the countries to identify single interventions capable of advancing the status of several component and/or core capabilities. For instance, the creation of a department of laboratory directly under the authority of the Ministry of Health was identified as one of the most effective strategies to leverage improvements regarding governance, network coordination, financing, legislation and policy pertaining to the laboratory sector.
3. Metrics including the private laboratory sector were difficult to measure in practice and were kept to the strict minimum in the final version of the LABNET scorecard.
4. The inclusion of sections dedicated to ‘priority diseases’ (core capability 9) and ‘laboratory network coverage and rapid response’ (core capability 3) bind the evaluation framework together through their cross-cutting relationship with all other laboratory key functions.

## Anticipated utilisation and benefit of the LABNET scorecard assessment

An important outcome of the regional GHSA consultation for laboratory strengthening in October 2015 was the recognition that adequate laboratory network capability to operate is required to support the GHSA and achieve compliance to IHR requirements.^7,41^ Hence, future enhancement strategies should yield measurable improvement of the laboratory network functionality. The LABNET scorecard assessment was specifically designed to fulfill this need. It can be used as a stand-alone assessment tool but preferably in conjunction with the WHO Joint External Evaluation Tool.^42^ The latter option will allow the collection of in-depth information on national laboratory network capabilities, in the context of a larger assessment of country preparedness for health security. Additionally, the LABNET scorecard assessment offers a good starting point to combine in-depth evaluation of more specific laboratory aspects, such as AMR surveillance capability^22^ or biosafety/biosecurity,^43^ at either system or facility level.

Gaps and weaknesses identified during the assessment will allow countries to prioritise areas with most urgent needs, and serve as the basis for the formulation of tailored plans for laboratory capacity strengthening under the GHSA framework. The success story of the SLIPTA/SLMTA programmes^11,12^ illustrates how tailored laboratory improvement plans based on outcomes from rounds of standardised assessments, can lead to measurable advancement toward laboratory (quality) standards. Similar to the approach described in the WHO ‘Better Lab for Better Health’ initiative in Eastern Europe and Central Asia,^44^ the data collected during the LABNET scorecard assessment will inform the development or revision of the national laboratory policy and/or strategic plan. The standardised assessment process will allow monitoring the progress of individual countries over time (as part of the monitoring and evaluation process). The comparison of data from different countries will facilitate the implementation of regional capacity building programmes according to an economy of scale approach.

LABNET scorecard assessment reports include clear visual representations of the status of each core capabilities at the country level or at the regional level, highlighting critical weaknesses for which additional resources are required. These figures can be used by laboratory system managers when advocating to their governments or to international stakeholders for the improvement of laboratory systems.

## Perspectives and conclusions

We have developed a new matrix for the comprehensive evaluation of national laboratory functionality. The LABNET scorecard complements the WHO Joint External Evaluation Tool^42^ for health system-wide assessments by providing laboratory-specific information with a high level of granularity. Following the principle that ‘what gets measured, gets done’, we propose that the LABNET scorecard can be one of the essential pieces of a phased approach to implementing/strengthening laboratory capability and capacity to assure access to quality clinical and public health functions, in support of the achievement of global health security goals The GHSA initiative^31^ is a unique opportunity for countries in Africa and elsewhere, to access resources including professional services of ASLM and APHL to overcome the barriers to strengthening laboratory networks.

**BOX 2:** Lessons Learnt.

• Chronic inadequate funding of national laboratory networks in sub-Saharan Africa has been a root cause of the inability of countries to detect, respond to and prevent disease outbreaks. Essential clinical and public health functions are insufficient thereby preventing effective response to of global health security threats.
• The LABNET scorecard is a matrix measuring national laboratory network functionality along a linear scale of increasing capability and across nine key dimensions necessary for detecting, preventing and responding to infectious threats. The LABNET scorecard is complementary to the WHO Joint External Evaluation tool measuring country preparedness to health threats according to the requirements of IHR 2005 and can provide the objective basis to support the case for adequate funding.
• The LABNET scorecard can be one of the essential pieces of a phased approach to implementing/strengthening laboratory capability and capacity to carry clinical and public health functions, in support of global health security goals for countries in need. Endorsement by World Health Organization Regional Office for Africa is foreseen.

Country buy-in is an essential prerequisite to adoption of the LABNET scorecard and can be facilitated by the endorsement of WHO regional offices. We propose that the LABNET scorecard be applied under the coordination and the leadership and normative organisation of the WHO and its regional offices, through international laboratory professional organisations such as ASLM and APHL. Such a framework can ensure efficiency and economy of scale of the intervention, in a similar fashion to the SLIPTA/SLMTA approach for implementing laboratory quality management systems in Africa.^12^ The possibility of countries using the LABNET scorecard as a self-evaluation tool to keep track of their own progress and guide their capacity building efforts is another additional benefit of this tool.
